# Structural covariance of superficial white matter in mild Alzheimer's disease compared to normal aging

**DOI:** 10.1002/brb3.252

**Published:** 2014-07-28

**Authors:** Cristian Carmeli, Eleonora Fornari, Mahdi Jalili, Reto Meuli, Maria G Knyazeva

**Affiliations:** 1 LREN, Department of Clinical Neuroscience, Centre Hospitalier Universitaire Vaudois (CHUV), University of LausanneLausanne, Switzerland; 2 Department of Radiology, Centre Hospitalier Universitaire Vaudois (CHUV), University of LausanneLausanne, Switzerland; 3 CIBM (Centre d'Imagérie Biomédicale), CHUV UnitLausanne, Switzerland; 4 Department of Computer Engineering, Sharif University of TechnologyTehran, Iran; 5 School of Electrical and Computer Engineering, RMIT UniversityMelbourne, Australia

**Keywords:** Connectivity, graph theory, interhemispheric asymmetry, magnetization transfer imaging, U-fibers

## Abstract

**Introduction:**

Interindividual variations in regional structural properties covary across the brain, thus forming networks that change as a result of aging and accompanying neurological conditions. The alterations of superficial white matter (SWM) in Alzheimer's disease (AD) are of special interest, since they follow the AD-specific pattern characterized by the strongest neurodegeneration of the medial temporal lobe and association cortices.

**Methods:**

Here, we present an SWM network analysis in comparison with SWM topography based on the myelin content quantified with magnetization transfer ratio (MTR) for 39 areas in each hemisphere in 15 AD patients and 15 controls. The networks are represented by graphs, in which nodes correspond to the areas, and edges denote statistical associations between them.

**Results:**

In both groups, the networks were characterized by asymmetrically distributed edges (predominantly in the left hemisphere). The AD-related differences were also leftward. The edges lost due to AD tended to connect nodes in the temporal lobe to other lobes or nodes within or between the latter lobes. The newly gained edges were mostly confined to the temporal and paralimbic regions, which manifest demyelination of SWM already in mild AD.

**Conclusion:**

This pattern suggests that the AD pathological process coordinates SWM demyelination in the temporal and paralimbic regions, but not elsewhere. A comparison of the MTR maps with MTR-based networks shows that although, in general, the changes in network architecture in AD recapitulate the topography of (de)myelination, some aspects of structural covariance (including the interhemispheric asymmetry of networks) have no immediate reflection in the myelination pattern.

## Introduction

Interindividual variations in regional structural properties of the cortex including regional volume, thickness, and surface area covary across the brain (Chen et al. 2008; He et al. 2008; Mechelli et al. 2005; Wu et al. 2012; Yao et al. 2010; reviewed in Alexander-Bloch et al. 2013a; Evans 2013). This population covariance in structural properties suggests that some of them are coordinated in groups of brain structures, that is, that they form networks. Components of such networks may be the distributed parts of functional systems. For instance, the hippocampal volume strongly covaries with that of the amygdala, and with those of the entorhinal, perirhinal, orbitofrontal, and parahippocampal cortices involved in the memory system (Bohbot et al. 2007). Areas implicated in language function are another example of a gray matter (GM) network (Zielinski et al. 2010).

The nature of population covariance in the normal brain can be partly explained by anatomical and functional connectivity (Gong et al. 2012; Alexander-Bloch et al. 2013b). The rest of covariance depends on shared genetic and developmental effects (Schmitt et al. 2008; Raznahan et al. 2011) and on learning and plasticity (Lv et al. 2008; Bermudez et al. 2009). Aging and accompanying neurological conditions may lead to changes in the patterns of structural covariance (Raz et al. 2005; Seeley et al. 2009). In this context, Alzheimer's disease (AD) is of special interest, since it affects distributed cerebral regions, which form an AD-specific spatial pattern (Buckner et al. 2005; Seeley et al. 2009; Knyazeva et al. 2010, 2013). As demonstrated by neurofibrillary pathology and structural atrophy, it is chiefly defined by the initial damage in medial temporal lobes, which spreads to the association cortices as the disease progresses (Braak et al. 2006).

This pattern of degeneration is also reflected in the demyelination of the brain. Animal models and human studies show a close association between AD-specific biomarkers and axonal demyelination (Desai et al. 2010; Mitew et al. 2010; Stricker et al. 2009: for review see Bartzokis 2011). Recently, we have demonstrated that the spread of demyelination in a typical precursor of AD – amnestic mild cognitive impairment and the demyelination topography of the superficial white matter (SWM) in mild AD patients – correspond to the AD-specific configuration (Fornari et al. 2012; Carmeli et al. 2013). The SWM is mainly composed of short association U-fibers. These fibers are formed by the axons of pyramids from layers III and V of the cortex. U-fibers leave the cortex, follow its folding within the underlying thin layer of the SWM, and re-enter the cortex at a distance of up to 30 mm (Schuz and Braitenberg 2002). In spite of their apparent importance as components of cortico-cortical networks that provide cascading connections between primary, sensory association, and multimodal areas, there is only scarce evidence supporting the U-fibers’ involvement in various functional processes and their changes in psychiatric and neurodegenerative diseases. Recent structural and diffusion MRI (magnetic resonance imaging) studies showed their contribution to the plastic reorganization of neural circuits after early blindness (Park et al. 2007) and their reduced structural integrity in multiple sclerosis (Miki et al. 1998), schizophrenia (Phillips et al. 2011; Nazeri et al. 2013), and autism (Shukla et al. 2011), in elderly relative to young people (Phillips et al. 2013), and in age-related impairment of gait (Srikanth et al. 2010).

Importantly, the demyelination of U-fibers detectable within the layer of SWM is among the early signs of neurodegeneration in AD. Moreover, the SWM demyelination correlates with cognitive decline even in mild AD (Fornari et al. 2012), suggesting a nearly immediate impact on the patient's state. Its progression forms a whole-brain AD-specific pattern, suggestive of changes at the network level. Such changes would be important for developing demyelination-based biomarkers of AD.

The structural covariance of the SWM has never been investigated since, as previously mentioned, the network studies were mostly based on GM properties. Thus, here we report the first attempt to apply the network theory to a characterization of the topology of cortical networks based on the structural properties of the SWM in elderly controls and AD patients. To this end, we use magnetization transfer imaging (MTI) that provides a myelin-sensitive contrast (Stanisz et al. 1999; Wozniak and Lim 2006). Indeed, postmortem studies show that MTI measurements strongly correlate with demyelination and axonal loss in the diseases associated with myelination abnormalities (Schmierer et al. 2007; Gouw et al. 2008). The independence of MTI from the spatial organization of fibers makes it a valuable technique for assessment of the SWM with its plentiful cross-oriented fibers.

The ultimate purpose of this article was to analyze the nature of SWM network changes in AD and their potential for understanding the underlying pathological process. To this end, we investigated how the networks obtained with graph-theoretical analysis map onto the SWM landscape, the latter being defined by the changes in myelin content in the posterior-to-anterior and left-to-right (inter-hemispheric) axes of the brain. Since these two levels of analysis are based on the same biological substrate, a comparison between them has the potential to suggest a plausible interpretation of the network data or, at least, to narrow down the search space for it.

## Methods

### Patients and control subjects

This study is based on the MTI data of 15 patients with probable AD and 15 control subjects. Previously, this sample was used for the analysis of demyelination of the SWM in AD (Fornari et al. 2012) and was a part of a larger sample, in which the topography of functional cortical connectivity was studied (Knyazeva et al. 2010). The patients were recruited from the Memory Clinic of the Neurology Department (CHUV, Lausanne). For the details of screening procedures and diagnosis assignment, see our recent reports (Knyazeva et al. 2010, 2013).

The AD group included six women and nine men (Table 1). The control subjects (nine women and six men) were volunteers (12 community-dwelling aged adults and three partners of AD patients). The patient and control groups differed neither in age nor in their gender. All but one participant in each group were right-handed. All the patients, caregivers, and control subjects gave written informed consent. All the applied procedures conform to the Declaration of Helsinki by the World Medical Association (2001) concerning human experimentation and were approved by the local Ethics Committee of Lausanne University.

**Table 1:** Demographic and clinical characteristics of AD patients and control subjects

Feature	AD patients	Control subjects	Statistical comparison
# of subjects	15	15	–
Gender M/W	6/9	9/6	*P* > 0.15
Age (years)	67.9 ± 10.5	64.5 ± 11.5	*P* > 0.4
MMSE	21.5 ± 4.0	28.9 ± 1.1	*P* < 0.001
CDR	0.8 ± 0.25	–	–

The second and third columns present group characteristics (mean +/− standard deviation). “W” stands for women, “M” for men. The fourth column presents *P*-values for the statistical significance of the two-sided between-group differences estimated by the Mann–Whitney-Wilcoxon test and the *χ*^2^ test for gender.

The clinical diagnosis was made according to the NINCDS–ADRDA criteria (McKhann et al. 1984) and confirmed by measuring the total hippocampal volume as a structural biomarker of AD. Cognitive functions were assessed with the Mini Mental State Examination (MMSE) and with a detailed standardized neuropsychological assessment scale carried out by the GRECO group for the French-speaking population (Puel and Hugonot-Diener 1996). The stage of dementia was determined according to the Dementia Rating Scale (CDR). For this analysis, we selected patients with mild dementia (CDR 0.5–1). In addition to the basic neuropsychological assessment, the severity of memory deficits considered to be typical early symptoms of AD was examined by means of the Mattis Dementia Rating Scale (Mattis 1976). Episodic verbal memory was evaluated using the 16-item test by Grober and Buschke (Grober and Buschke 1987). Episodic nonverbal memory was assessed through the shape test from the “Doors and People” test (Baddeley et al. 1994). Access to semantic knowledge was tested by means of Lexis test (De Partz et al. 2002).

Complete laboratory analyses and diagnostic neuroimaging (CT or MRI) were performed in order to rule out cognitive dysfunctions related to the causes other than AD. The exclusion criteria were severe physical illness, psychiatric or neurological disorders associated with potential cognitive dysfunction, other dementia conditions (frontotemporal dementia, dementia associated with Parkinsonism, Lewy body disease, pure vascular or prion dementia, etc.), alcohol/drug abuse, and regular use of neuroleptics, antidepressants with anticholinergic action, benzodiazepines, stimulants, or *β*-blockers.

Control subjects underwent a brief clinical interview and the MMSE, to confirm the absence of cognitive deficits, of the use of psychoactive drugs, and of diseases that may interfere with cognitive functions. Only individuals with no cognitive complaints and an MMSE score ≥28 and ≥26 for those with a low level of education (primary or secondary school without, or with short professional training) were accepted as controls. All control subjects underwent a brain MRI.

**Figure 1:**
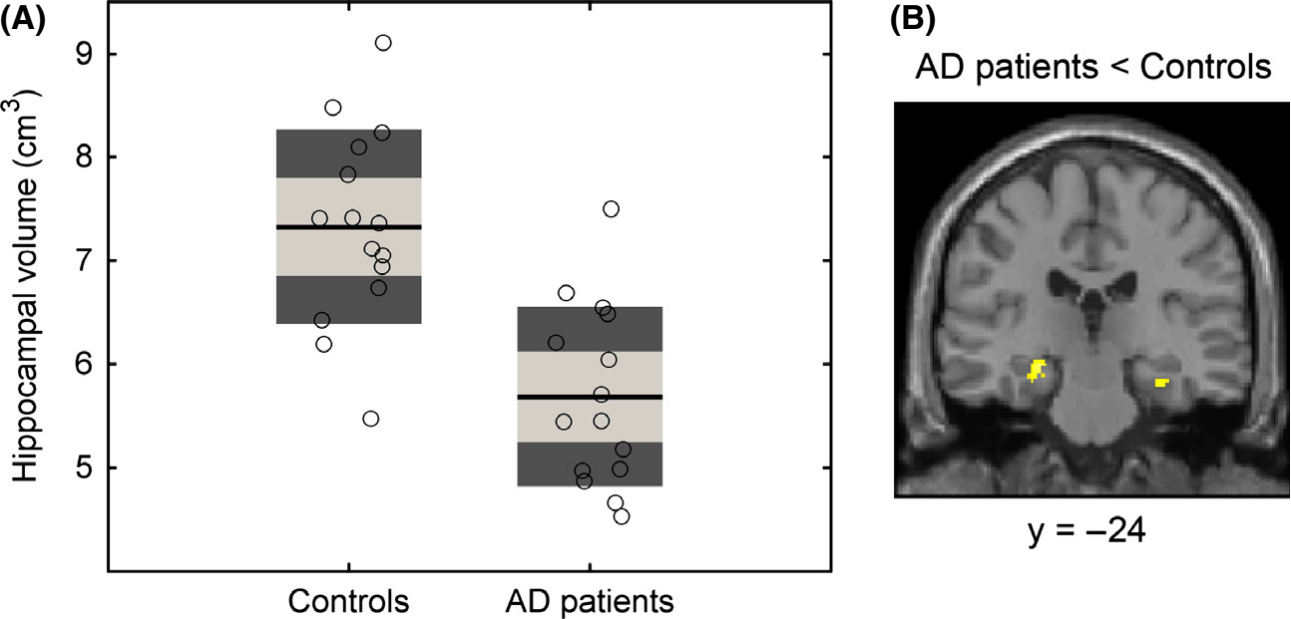
Hippocampal volume in AD patients and control subjects. (A) The total (left hemisphere + right hemisphere) volume of the hippocampus is shown for the control and AD groups. For each group, the estimated individual values are shown with empty black-bordered circles. The black lines represent the group mean, the light gray boxes represent the interval spanned by the mean ± 1 SD, and the dark gray boxes the mean ± 1.96 SD. The between-group contrast (AD < Controls) is significant at *P* < 0.001 (GLM with total intracranial volume, age, and gender as covariates). (B) The coronal slice (*y* = −24) shows the clusters of voxels (yellow) in the hippocampi where we found a loss of gray matter volume in the AD group (*P* < 0.05, cluster level FWE-corrected). For presentation purposes, the SPM is overlaid on the T1-weighted image of the population average (an output of the DARTEL algorithm) translated into the MNI space.

Following recent recommendations of the National Institute on Aging and Alzheimer's Association workgroups (Jack et al. 2011; McKhann et al. 2011), we measured the total hippocampal volume of both patients and controls (Fig. 1), since a smaller hippocampus is a structural biomarker of AD. In the AD group, the volume of the hippocampus turned out to be 22% lower than in control subjects at *P* < 0.001 (Fig. 1A). A voxel-based morphometry analysis with SPM8 confirmed this result. We found two significant (*P* < 0.05 FWE-corrected at a cluster level) clusters located in the left and right hippocampi, where in AD patients GM volume was lower than in control subjects (Fig. 1B; for methods see Data S1).

### Magnetic resonance imaging

All the patients and controls were scanned in a 3 Tesla Philips Achieva scanner (Philips Medical Systems, Best, the Netherlands). The protocol included a sagittal T1-weighted 3D gradient-echo sequence (MPRAGE, 160 slices, 1 mm^3^, isotropic voxels) as a basis for segmentation. We performed MTI by running a gradient-echo sequence (FA 20, TE 10, matrix size 192*192, pixel size 1.3*1.3 mm, 52 slices (thickness 2.5 mm), spatial resolution of ∼4 mm^3^) twice, first with and then without an MT saturation pulse. We used a Gaussian MT prepulse with a duration of 7.68 ms, FA 500, and a frequency offset of 1.5 kHz. The entire protocol (for detailed description see Fornari et al. 2012) lasted 22 min.

MTI acquisitions were coregistered on the high-resolution T1 acquisition without resampling, thus maintaining the original resolution of each modality. For every intracranial voxel (spatial resolution of ∼4 mm^3^), we calculated the magnetization transfer ratio (MTR) as follows:


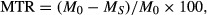
(1)

where *M*_S_ represents the intensity of the signal in a voxel with saturation, *M*_0_ without saturation. The ratio indicates the percentage loss of signal intensity attributable to the MT effect. Since this effect mainly depends on the myelin concentration (Stanisz et al., 1999), a decrease in MTR values is considered to be a sign of demyelination and/or a loss of axons. In order to minimize the effect of noise, only voxels with MTR > 10% were included in the analysis. Image processing was performed using SPM8 and ad-hoc routines developed in the Matlab 7.1 environment.

#### Segmentation of gray matter, white matter, and superficial white matter

For each subject, the high-resolution anatomical T1 images of the brain were segmented into GM, white matter (WM), and CSF using the unified segmentation algorithm in SPM8.

In order to select the SWM below the cortex, we first defined its outer surface as the external surface of the WM mask obtained by thresholding the WM probability map at *P* > 0.95. The selected high level of significance allowed us to minimize the partial volume effect in the selected voxels. Then, the so-defined WM mask was subjected to an iterative erosion process ending at a depth of 3 mm. The inner surface of the SWM was defined as an external surface of the eroded WM mask. Therefore, the volume between the inner and the outer surfaces constituted the 3-mm-thick layer of WM below the cortex, that is, the SWM. All these operations were performed by in-house-made routines in Matlab.

Each hemisphere was then divided into 39 ROIs, mainly corresponding to Brodmann areas (BA), and the mean MTR value was calculated for the 3-mm-thick SWM underneath each ROI by dilating the cortical ROI towards the SWM until they intersect (for details see Fornari et al. 2012). All these operations were performed in the native space for each subject. Mean MTR values for each group and for each ROI were displayed with the Caret software (http://neuro.debian.net/pkgs/caret.html) on a mesh representing the surface of the GM/WM boundary of a standard MNI (Montreal Neurological Institute) brain. For additional details of MT imaging, segmentation, and analysis see (Fornari et al. 2012).

#### MTR-based parameters and statistics

For each ROI, we computed a laterality score as (L − R) / (L + R), where L and R stand for the MTR values of a pair of homotopic areas in the left and right hemispheres, respectively. For statistical inference, we built a General Linear Model (GLM) with the laterality indices of the two groups (controls and AD patients) as dependent variables, and age and gender as nuisance variables. The distributions of the *t*-statistics for each contrast of interest (a one-sample *t*-test for within-group and a two-sample *t*-test for between-group contrasts) were estimated through 10,000 permutations. *P*-values were corrected for multiple comparisons through the linear step-up false discovery rate (FDR) (Benjamini and Hochberg, 1995). We considered an FDR-corrected *P* < 0.05 significant.

### Graph-theoretical analysis

#### Network estimation

The MTR data from all the ROIs were embedded in a graph, where nodes represent the areas, and edges represent the statistical associations between them. In contrast to previous studies, which reported a seed-based inference, namely, the associations between a preselected area and remaining areas (Mechelli et al. 2005), here we consider the whole set of associations among the areas of interest.

While providing information about the entire network, our approach has to cope with a difficult estimation problem in a statistical setting, where the available number of samples is much lower than the number of variables or dimensions. Indeed, the statistical estimation of associations (i.e., the network model or Gaussian graphical model) is based on partial linear correlations, whose traditional estimators are applicable only if the number of samples (*n*) is larger than the number of nodes (*k*) (Whittaker 1990). This is not our case, since for each group we have *n* = 15 and *k* = 78. To overcome this issue, we applied a shrinkage-based estimator of the partial correlation matrix (Schäfer and Strimmer 2005). Shrinkage is a regularization technique for ill-posed estimation problems. It is based on additional information, which is not used by standard estimators (e.g., maximum likelihood). To this end, the shrinkage estimator takes advantage of a target estimate. In our case, a heteroscedastic diagonal covariance matrix corresponding to a network/graphical model made of isolated nodes was the target. In practice, the shrunken covariance matrix is a weighted average of the standard and target estimates, which average has a minimum mean squared error, and is well-conditioned and positive-definite (Schäfer and Strimmer 2005). Given the two latter properties, the matrix of partial correlations can be reliably computed from the inverse of that shrunken covariance matrix. Using Σ to signify the *k* × *k* estimated covariance matrix, the partial correlation matrix is computed as


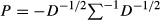
(2)

where *D* is a diagonal matrix obtained by extracting the diagonal elements of Σ^−1^. Note that for good estimation of partial correlations, a well-conditioned Σ is crucial.

It is important to note that, compared to full correlations, partial correlations allow better distinctions to be made between direct and indirect associations (Walker et al. 2010; Jalili and Knyazeva 2011; Smith et al. 2011).

#### Statistical inference of networks

We used statistical inference to determine the correlation structure for (1) the AD group and the control group separately; (2) AD vs. control group; and (3) left vs. right hemisphere. For *i)*, we modeled the probability of a partial correlation or edge being null through the local false discovery rate (*lfdr*) (Efron 2004; Schäfer and Strimmer 2005). The *lfdr* corresponds to the posterior probability of an edge to be null given the estimated *p*_*ij*_ (an element of the matrix obtained with Eq. 2). In formulae


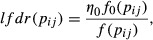
(3)

where *f*_*0*_ is the null distribution of the *p*_*ij*_ provided in (Hotelling 1953), *η*_0_ is the unknown proportion of null partial correlations, and *f* is the distribution of the *p*_*ij*_. The distribution *f* can be modeled as a mixture of two distributions (Efron 2004), namely


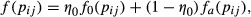
(4)

where *f*_*a*_ is the (unknown) distribution of estimated partial correlations assigned to truly nonnull edges. The unknown parameters can be estimated from the empirical distribution of the set of *p*_*ij*_, and an efficient estimation procedure is implemented in the R-package fdrtool (Strimmer 2008).

The *lfdr* directly takes into account the multiplicity of null hypotheses, and warrants a Bayesian approach to detecting nonnull partial correlations. To this end, partial correlations with *lfdr* < 0.2 are deemed *highly* significant ones (Klaus and Strimmer 2013). To establish the nonnull partial correlations, we applied a threshold *lfdr* < 0.2, thus obtaining partial correlations with a very high positive predictive value (>0.9), i.e., very likely reflecting true associations (Schäfer and Strimmer 2005). Linear effects of age and gender were removed by including them in the computation of Eq. 2 as additional variables (*k* = 80). For further analysis, only partial correlations related to BA-based regions were retained.

To analyze (2) and (3), we applied a Fisher transformation to the partial correlations, computed z-scores from the relevant pair of samples, and, finally, calculated *lfdr* from the z-scores. In this case *f*_*0*_ (Eqs. 3 and [Disp-formula m4]) was the Gaussian distribution. We verified this assumption by running a Jarque–Bera test (Jarque and Bera 1987) on the z-scores (*P*-value > 0.5).

The correlations (edges) between the two groups or hemispheres associated with *lfdr* < 0.2 were considered significantly different.

#### Network topological properties

The structure of MTI-based networks was analyzed with two approaches: a macro-network and a conventional topological one. For the macro-network approach, we applied a mapping of the 78 nodes across frontal, temporal, parietal, occipital, and paralimbic lobes (Table 2). To summarize intra- and inter-lobar associations, we employed a framework developed in the context of brain functional integration–segregation theory (Tononi et al. 1994). If *N*_1_ is a group of nodes corresponding to a certain ROI, the amount of association or correlation in that region is computed through Gaussian entropy


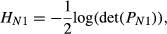
(5)

where *P*_*N*1_ denotes the partial correlation matrix computed with Eq. 2, restricted to the nodes in *N*_1_. *H*_*N*1_ provides the measure of the distance from zero correlation.

**Table 2 tbl2:** Clusters of ROIs for macro-network mapping

Region/Lobe	Brodmann areas
Frontal	4, 6, 8, 9, 10, 13, 44, 45, 46
Parietal	1+2+3, 5, 7, 39 40, 43
Paralimbic	11, 23, 24 + 33, 25, 27, 28, 29+30, 31, 32, 34, 35, 36, 38, 47
Temporal	20, 21, 22, 37, 41, 42
Occipital	17, 18, 19, Cuneus

The amount of correlation between two regions *N*_1_ and *N*_2_ is computed through their mutual information, namely


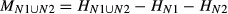
(6)

To adapt graphical representations for this analysis and to allow a comparison between AD patients and controls, we ranked the intra- and inter-lobar associations into three levels (weak, medium, and strong) via a *K*-means algorithm.

In the conventional topological approach, we studied various metrics introduced in the complex network field including node centrality, global efficiency, local efficiency, and modularity (Boccaletti et al. 2006; Bullmore and Sporns 2009). Let us denote by *A* = {*a*_*ij*_} the adjacency matrix of a binary undirected network *G*, where an element *a*_*ij*_ is nonnull, if an edge between nodes *i* and *j* exists. The degree of a node *i* is defined as


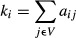
(7)

where *V* is the set of all nodes of *G*. The degree of a node is considered a measure of node centrality. Nodes with a centrality value lying in the upper quartile of the integrated (see below for details) node degree distribution were defined as hubs (Sporns et al. 2007).

*Global* efficiency is equivalent to the inverse of the harmonic mean of the length of the shortest paths in each pair of nodes in the network, whereas *local* efficiency is restricted to the topological first neighbors. In other words, global efficiency is related to global interconnectedness, whereas local efficiency is related to local interconnectedness (Latora and Marchiori 2001). In formulae, efficiency is defined as


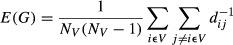
(8)

where *N*_*V*_ is the number of the nodes of network *G*, and *d*_*ij*_ is the length of the shortest path between nodes *i* and *j*. The definitions of global and local efficiency follow as


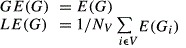
(9)

where *G*_*i*_ is the sub-network composed of the first-order (topological) neighborhood of node *i*.

A module is defined as a group of nodes connected by a larger number of within-group edges than between-group edges (i.e., connecting the group with the rest of the node groups). The *Q* index quantifies the degree of modularity of *G* and is calculated as in (Newman 2006).


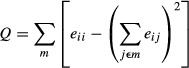
(10)

where *m* is the predetermined set of modules with nonoverlapping nodes, and *e*_*ij*_ stands for the proportion of all links connecting nodes in module *i* with those in module *j*. *Q* quantifies the degree of a modular structure with *m* modules, in which the larger the *Q,* the more modular the network.

We investigated these four metrics over a range of densities chosen according to the *lfdr* statistics such that 0.2 < *lfdr* < 0.5 (see section *Statistical inference of networks*). To this end, the partial correlation matrix computed with Eq. 2 was thresholded in that *lfdr* range. As formulated in (Klaus and Strimmer 2013), *lfdr =* 0.5 represents a principled (in a Bayesian sense) estimation of the boundary between the null hypothesis and the alternative hypothesis. Practically, partial correlations associated with *lfdr* < 0.5 are considered significant nonnull correlations, while those with *lfdr* < 0.2 are deemed *highly* significant.

In particular, we applied density integration, recently proposed by (Ginestet et al. 2011). To this end, the metrics were computed at a chosen range of densities and then integrated over the range. This amounted to an averaging since we assumed that the different densities were equally likely. Numerical computations of the network metrics were performed with the BCT toolbox (https://sites.google.com/site/bctnet/).

To compare the metrics *GE*, *LE,* and *Q* between AD patients and control subjects, without being able to assume a distribution of the three metrics, we applied a non-parametric permutation technique. Namely, the individual values for *GE*, *LE*, and *Q* were randomly shuffled 10,000 times. At each shuffle, we computed partial correlations and the three network metrics for each density, and integrated over densities. The absolute value (two-sided test) of each difference from the permuted data was then compared to the respective difference from the original data (AD patients vs. control subjects), and *P*-value was estimated as the fraction of permutations showing a larger difference.

We also compared the three metrics in a different way. As in (Achard and Bullmore 2007; van Wijk et al. 2010; Joudaki et al. 2012), we constrained the two networks so as to give them the same density (i.e., the same percentage of nonnull edges) and binarized them by assigning 0 to null edges and 1 to nonnull edges. The metrics of the two networks were then compared density-wise for the chosen ranges. While this approach avoids comparing networks with an unbalanced number of edges and disentangles the effect of topology from the effect of difference in edge density, it requires a correction for multiple comparisons.

## Results

### Intra- and inter-hemispheric MTR landscapes in elderly controls and AD patients

**Figure 2:**
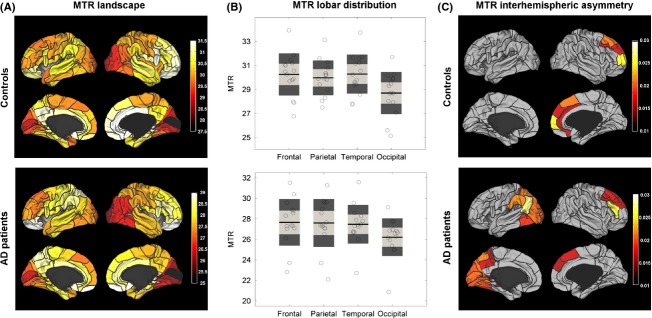
Landscapes of myelination of superficial white matter in elderly controls and AD patients. (A) The mean areal MTR values are shown with 3D rendering in the lateral and medial views of the two hemispheres. The color-bars represent the raw MTR values, gray represents regions where BA are not defined. Rendering and display of the maps have been obtained with Caret software (http://www.nitrc.org/projects/caret/). (B) The lobar MTR values (left hemisphere + right hemisphere) are shown for controls (top) and AD patients (bottom). For each group, the individual values are shown with empty black-bordered circles. The black lines represent the group mean, the light gray boxes the mean ± 1 SD, and the dark gray boxes the mean ± 1.96 SD. (C) The interhemispheric asymmetry of MTR values is color-coded in controls (top) and AD patients (bottom) according to the value of laterality index. Note that for presentation purposes, we show only regions with a nonzero laterality index (*P* < 0.05, uncorrected). Gray regions refer to insignificant interhemispheric differences in MTR values (*P* > 0.05, uncorrected). Both controls and AD patients demonstrate a rightward asymmetry in the prefrontal regions. The parietal and occipital ROIs show an asymmetry of SWM only in the AD group.

By “MTR landscape”, we mean the SWM map with its distinct regional features manifested through regional MTR values. The intra-hemispheric landscapes showed a tendency of posterior-to-anterior increase in MTR values in the control and AD groups (Fig. 2A). To estimate it, we contrasted the mean lobar values of MTR across the frontal, parietal, temporal, and occipital lobes (Fig. 2B, Table 2). Although the contrasts did not survive FDR correction, occipital MTR showed a tendency to be lower than frontal MTR (*P* < 0.05, uncorrected) in both groups.

The interhemispheric asymmetry in controls was significant for the anterior prefrontal cortex (BA 10) at *P* < 0.05 (FDR-corrected) with the higher MTR values in the right hemisphere (Fig. 2C). Similar trends were characteristic for the frontal eye field (BA 8), the dorsolateral prefrontal cortex (BA 9), and the anterior cingulate (BA 32) at *P* < 0.05 (uncorrected). In AD patients a rightward asymmetry of the prefrontal SWM was significant in the frontal eye field (BA 8) and the dorsolateral prefrontal cortex (BA 46) at *P* < 0.05 (FDR-corrected), but did not survive correction for multiple comparisons in BA 9. The postcentral ROIs showed an asymmetry of SWM only in the AD group. Specifically, we found widely spread leftward asymmetry in the parietal and occipital areas (BAs 5, 7, 17, 18, 31) at *P* < 0.05 (FDR-corrected). A similar tendency (*P* < 0.05, uncorrected) could be seen in the posterior temporal and medial occipital regions (BAs 19, 28–30, 37, 39–40, and the cuneus). However, in the between-group comparison, only the inferior temporal gyrus and the anterior part of the fusiform gyrus (BA 37) showed a propensity for higher laterality scores in the AD group (*P* < 0.05, uncorrected).

### MTR-based networks in elderly controls and AD patients

#### General characteristics

**Figure 3:**
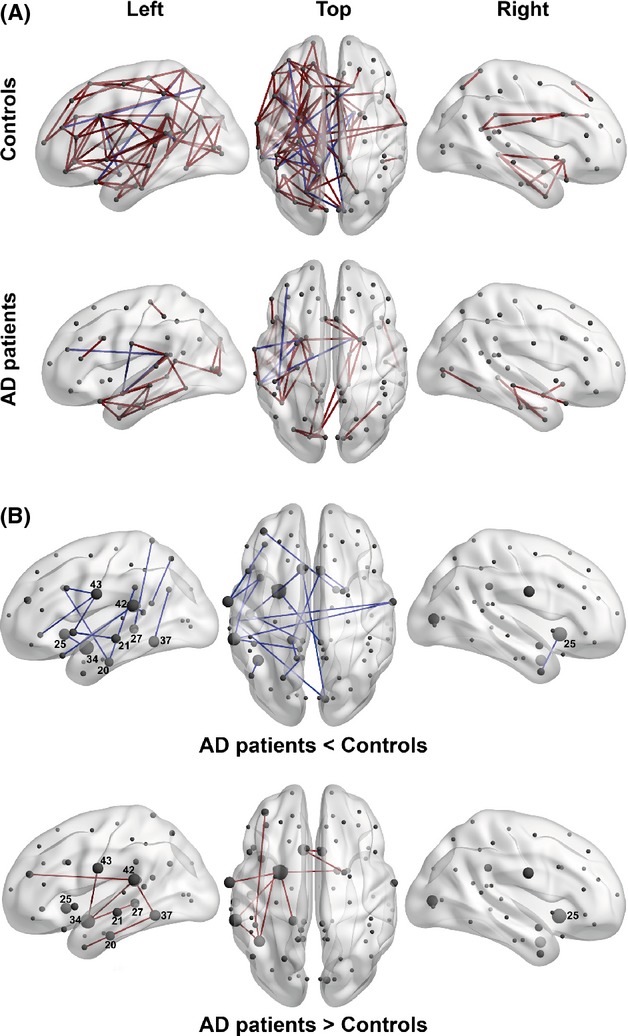
The MTR-based covariance networks in controls and AD patients. (A) The networks are rendered on the 3D smoothed brain of the ICBM152 template with the BrainNet Viewer (http://www.nitrc.org/projects/bnv/). They are presented in the left lateral, top, and the right lateral views of the brain for controls (top row) and AD patients (bottom row). Nodes are designated as gray circles located at the centers of mass of each ROI. Significant edges (*lfdr* < 0.2) are drawn in red for positive partial correlations and in blue for negative partial correlations. (B) The edges significantly different between elderly controls and AD patients (*lfdr* < 0.2) are rendered as in Fig. 3A. A node size corresponds to the degree of difference (i.e., to the number of edges significantly different in the AD compared to the control group). The nodes labeled with the associated Brodmann area number in white have degrees larger than two. The blue edges are present in controls, but not in AD patients (top row), while the red edges are present in AD patients, but not in controls (bottom row).

The network inferred for the control subjects shows 107 (∼3.6% of total number edges) significant edges (*lfdr* < 0.2) that mostly connect the nodes of the left hemisphere (Fig. 3A; Table 3). The network deduced for AD patients includes 42 (∼1.4% of total number of edges) edges (*lfdr* < 0.2), also located largely in the left hemisphere. In both groups, about 90% of edges are positive correlations between nodes.

**Table 3:** Characterization of inferred networks: edges

Group/Location	Left Hemisphere	Right Hemisphere	Interhemispheric
Controls (#positive/#negative)	67 edges/7 edges	17 edges/0 edges	10 edges/6 edges
AD (#positive/#negative)	26 edges/3 edges	9 edges/0 edges	3 edges/1 edge

The table shows the number of significant edges (*lfdr* < 0.2) for control and AD groups according to their gross topology, namely connecting nodes within the left or right hemispheres only, or connecting nodes of different hemispheres. Finally, the number of edges associated with positive or negative partial correlation is reported. The average strength of edges was about 0.109 for controls and 0.120 for AD. There were disconnected nodes in both populations: 35 in controls (six in the left hemisphere and 29 in the right hemisphere) and 54 in AD (22 in the left hemisphere and 32 in the right hemisphere).

Statistical analysis of partial correlations showed that, compared to controls, AD patients demonstrate both increased and decreased inter-areal correlations. Specifically, AD networks lost 20 and gained 10 edges (Fig. 3B, Table 4). Nearly all of them represent edges connecting the left temporal nodes. However, the lost edges (2/3 of all changed correlations) predominantly reflect alteration in covariance between temporal areas and relatively distant regions located in the frontal and parietal lobes and in the opposite hemisphere, whereas the new edges are concentrated within the temporal lobe, where they show strengthened covariance among areas located in the parahippocampal and fusiform gyri, and among the lateral temporal areas.

**Table 4 tbl4:** Differences in edges between controls and AD patients

Comparison	Left intrahemispheric	Right intrahemispheric	Interhemispheric
AD < Controls	12 edges	1 edge	7 edges
AD > Controls	7 edges	0 edge	3 edges

The number of significant edges (*lfdr* < 0.2) different between control and AD groups is shown. The differences refer to the edges that are present in one group but not in the other independently from the sign of partial correlation. The differences are reported separately for intra- and inter-hemispheric edges.

**Figure 4:**
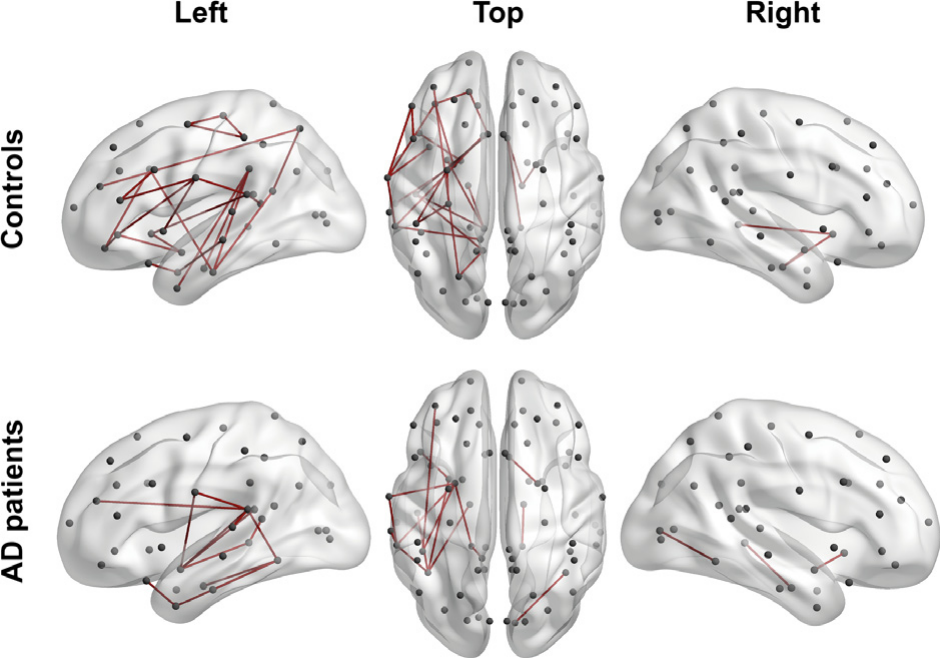
Interhemispheric network asymmetry in elderly controls and AD patients. The edges significantly different (*lfdr* < 0.2) between the left (LH) and right (RH) hemispheres are rendered as in Fig. 2. The edges are drawn in the LH, if corresponding partial correlation values are higher in the LH, and in the RH, if the opposite is true.

Analysis of the interhemispheric asymmetry of edges provided 26 significantly different edges (LH > RH for 23 and LH < RH for 3 of them) in the control group, and 16 edges (LH > RH for 13 and LH < RH for 3) in the AD group (Table 2013a). In controls, the LH > RH edges were distributed in the temporal, parietal and frontal lobes, while in AD patients they were mostly localized in the temporal lobe (Fig. 4).

**Table 5:** Edge asymmetry in controls and AD patients

Group/Comparison	LH > RH	LH < RH
Controls	23 edges	3 edges
AD patients	13 edges	3 edges

The table shows the number of significant edges (*lfdr* < 0.2) that differ between the left (LH) and right (RH) hemispheres in the control and AD groups. The interhemispheric difference was computed independently from the sign of partial correlations.

#### Topological properties

**Figure 5:**
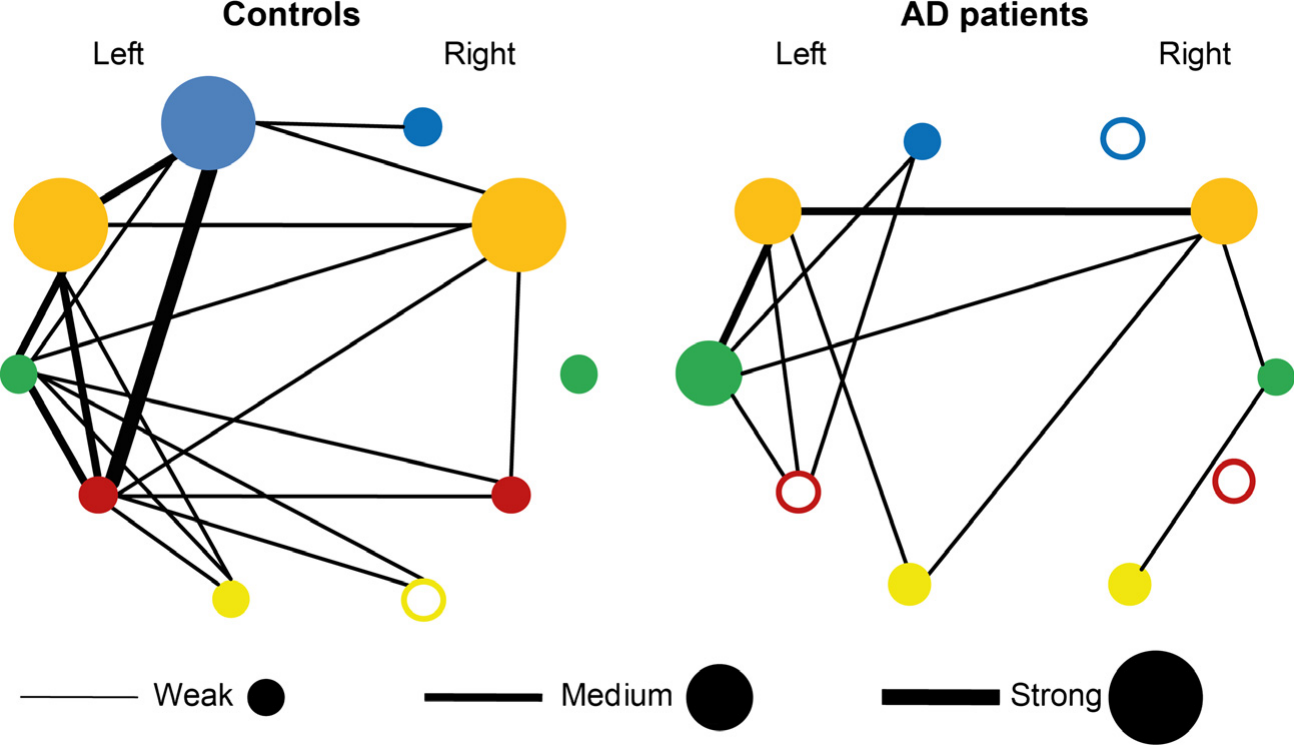
Macro-networks in elderly subjects and AD patients. The networks including the frontal (blue), paralimbic (orange), temporal (green), parietal (purple), and occipital (yellow) regions are drawn for controls and AD patients. Nodes represent intraregional connectivity (correlation). The empty nodes stand for insignificant intraregional correlation, while colored nodes correspond to significant intraregional correlations. In so doing, the radius of a colored node corresponds to an intracorrelation level (weak, medium, or strong). Edges represent interregional connectivity (correlations) and their thickness matches one of the three levels described for the nodes.

With macro-network mapping, we categorized correlations into relatively short-range (mostly intra-lobar) and long-range (inter-lobar and inter-hemispheric) connections (Table 2). As can be seen from Fig. 5, intralobar connections are predominantly symmetric with the exception of the frontal lobe in the control group and the temporal lobe in the AD group. All but one (temporal) ROI lose their internal covariance in AD; this is especially noticeable in the paralimbic regions of both hemispheres and the left frontal lobe, which show the strongest intracorrelations in the control subjects. In agreement with Fig. 4, both macro-networks show leftward asymmetry, which emerges mainly at a level of interlobar connections. Remarkably, the right hemisphere ROIs correlate with the homo- and hetero-topic regions of the left hemisphere rather than intrahemispherically. Most of interlobar correlations are weakened in the AD group, except those connecting paralimbic regions of the opposite hemispheres. This loss is especially pronounced for the connections of the left frontal and parietal areas, the strongest in the control group.

**Figure 6:**
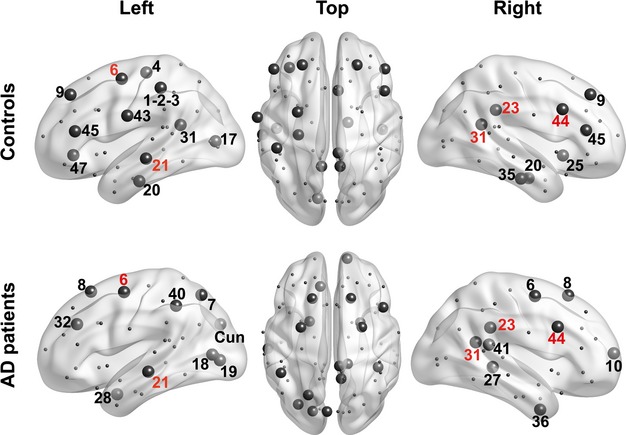
Network hubs in AD and control subjects. Hubs in control and AD subjects (top and bottom row, respectively) are drawn as large circles. They correspond to the nodes with an integrated node degree value lying in the upper quartile of the distribution. The numbers denote the Brodmann areas where corresponding nodes are located. Hubs common to the control and AD subjects are designated with red numbers, while group-specific hubs are designated with black numbers.

In the controls, we found 11 hubs in the left hemisphere and eight hubs in the right hemisphere (Fig. 6). Out of the total number of hubs, eight are located in the prefrontal areas, seven hubs in the paralimbic and temporal regions, and four hubs in the primary motor, somatosensory, gustatory, and visual areas (Table 6). AD patients have 10 hubs in the left and nine hubs in the right hemisphere, but only seven of them are the same as in the controls, including posterior cingulate, parahippocampal, and some temporal hubs. The AD group lost 12 hubs, the majority of which (seven) belong to the frontal lobe. Remarkably, all four hubs located in the primary cortical areas are also absent in the patients. Among the hubs acquired by AD group, there are five posterior hubs (all in the left hemisphere), four prefrontal, and three temporal.

**Table 6:** Hub topography

Group	Left hemisphere	Right hemisphere
Controls	1-2-3, 4, 6, 9, 17, 20, 21, 31, 43, 45, 47	9, 20, 23, 25, 31, 35, 44, 45
AD patients	6, 7, 8, 18, 19, 21, 28, 32, 40, Cuneus	6, 8, 10, 23, 27, 31, 36, 41, 44

The table shows the numbers of Brodmann areas identified as hubs, that is, the nodes with degree values belonging to the upper quartile of node degree distribution (for AD and Control subjects, respectively).

**Figure 7:**
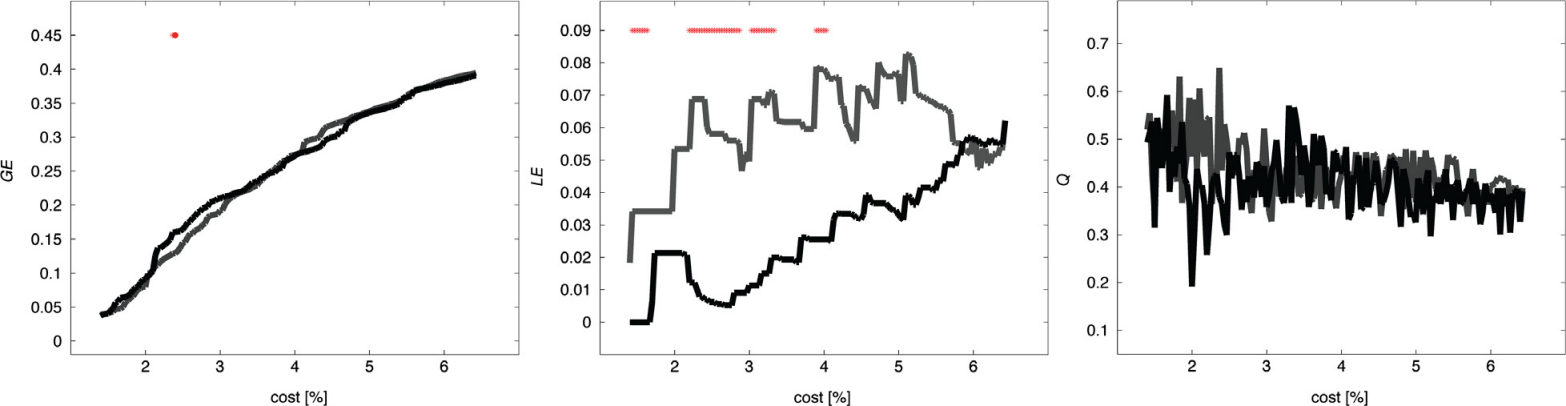
Network topology in AD and control subjects. The curves show the global efficiency *GE* (left panel), local efficiency *LE* (middle panel), and modularity index *Q* (right panel) as a function of density (percentage of nonnull edges) in AD patients (black) and controls (gray). Red stars show cost values, for which there is a significant difference between the AD and the control groups (*P* < 0.05, uncorrected for multiple comparisons).

The analysis of network topology showed that mild AD patients tended to have a decreased local efficiency (*LE*) at *P* < 0.05 (uncorrected) for several density ranges, while there were virtually no significant between-group differences in the modularity (*Q*) and global efficiency (*GE*) (Fig. 7). Within the framework of the integrated-density approach, these three metrics did not show significant differences between the controls and the AD group (*P* > 0.05).

## Discussion

Here, we report our results at two levels of analysis, namely (1) SWM myelination maps based on regional MTR values and (2) the structural networks that they form in elderly people and AD patients. The largest network changes due to AD are concentrated in the left hemisphere, which is characterized by the most significant reduction in myelin content of SWM (Fornari et al. 2012). These changes include both lost edges, which tend to connect temporal areas to remote intra- and inter-hemispheric regions or areas within lobes other than the temporal ones, and newly gained edges, which are predominantly limited to the temporal and paralimbic regions already affected by neurodegeneration in mild AD. Another finding is a striking leftward asymmetry of the intrahemispheric patterns of MTR-based regional covariance in healthy elderly subjects and AD patients not mirrored by an interhemispheric asymmetry of SWM maps. Indeed, the topography of SWM myelination in both groups is characterized by a weak rightward prefrontal asymmetry, supplemented in AD patients by a leftward posterior asymmetry. In the following discussion we consider the probable nature of the network level changes by comparing them with the spatial pattern of (de)myelination. We begin with a discussion of the advantages and limitations of the novel statistical technique that we applied here.

### Shrinkage estimator in clinical neuroscience

Recent advances in data acquisition techniques have led to the generation of massive datasets in many fields of science. These datasets (e.g., MRI data in neuroscience) are often characterized by high dimensionality and a relatively low number of samples (subjects), which hinders an application of classical statistical tools. In particular, widely used standard covariance and correlation estimators are not suitable for such datasets. For instance, maximum likelihood estimators are optimal only for a very large number of samples (Schäfer and Strimmer 2005).

In this study, to mitigate the effect of a small sample size while estimating a high-dimensional covariance, we applied the shrinkage technique introduced in (Schäfer and Strimmer 2005). This shrinkage covariance estimator provides well-conditioned and positive-definite covariances, thus allowing an estimation of Gaussian graphical models through inverse covariances (Whittaker 1990). Applied to simulated data, this estimator was shown to perform better than competing shrinkage techniques (e.g., penalized regression) in terms of sensitivity and positive-predictive value. Notably, it provides inference of Gaussian graphical models with very high positive-predictive value (Schäfer and Strimmer 2005). Due to the latter feature, any edge detected as significant corresponds to a true edge with very high probability. However, as expected, those statistical properties of the estimator fade away, when the ratio n/k (n samples and k dimensions, see Section *Network estimation*) becomes too small.

Below, we discuss some properties of SWM covariance networks and their differences between AD patients and controls and between the hemispheres obtained by means of the shrinkage estimator applied to relatively small samples. Yet we refrain from inferring whether the networks in these populations have a random or meaningful (i.e., small-world or scale-free) topology (Evans 2013). To address this question one needs to overcome two limitations inherent to the sample analyzed in this work: the small number of nodes (here, brain regions) and of samples (here, subjects). It is difficult to test small-worldness or scale-freeness if there are less than a few hundred nodes, since, in this case, inferred topological properties may depend on poor spatial sampling (Bialonski et al. 2010) and, thus, be biased. Increasing the number of nodes in the same number of subjects (due to the natural limitations of clinical samples) would result in decreasing the n/k ratio, so lowering or even fully losing the power of inference (Schäfer and Strimmer 2005).

### SWM maps and networks in elderly normals and AD patients

Early MTI studies, which analyzed regional WM including its deep and superficial components, reported the highest MTR values in the corpus callosum, followed by the association fibers of the neocortex, and further followed by the subcortical structures with myelinated fibers (Mehta et al. 1995; Silver et al. 1997). A more recent study (Armstrong et al. 2004) demonstrated a similar descending order of regional MTR values: the corpus callosum, cingulate, deep WM, brain stem, subcortical nuclei, and cerebellum. Moreover, in the prefrontal lobe, MTRs were higher than in the posterior frontal lobe, as well as in the lateral aspect of the temporal lobe compared to its medial aspect (Armstrong et al. 2004). Combined with the posterior/inferior-to-anterior/superior gradient of the WM maturation (Colby et al. 2011), such a distribution of the MTR values suggests that prolonged development of the WM in the anterior/superior regions results in their greater myelination, emphasizing the role of myelination in life-long plasticity.

Here we made an attempt to estimate whether a similar gradient exists for the SWM. The SWM is predominantly composed of short association fibers (U-fibers) connecting locations within the same area and/or adjacent gyri (Schuz and Braitenberg 2002; Oishi et al. 2011; Catani et al. 2012). They leave the cortex but follow its folding within the underlying thin layer and then reenter the cortex at a distance of up to 30 mm. In normal middle-aged individuals, the SWM is virtually free of lesions due to being vascularized with both deep and cortical arteries (Wen and Sachdev 2004). Yet, with age, healthy adults show reduced integrity of the SWM, varying from pronounced in the prefrontal regions to faintly detectable in the posterior and ventral regions of the hemispheres (Phillips et al. 2013).

We could demonstrate a trend of the myelination gradient only with the anterior MTR values higher than the posterior MTR values in both groups. The highest susceptibility of prefrontal SWM to the effects of aging can account for the nonsignificant posterior-to-anterior gradient.

In mild AD patients, as we showed recently (Fornari et al. 2012), MTR-based SWM maps clearly demonstrate an AD-specific pattern of demyelination, with the left medial temporal lobe (MTL) as the most affected region. Moreover, regional MTR values correlate with MMSE, language, and memory tests, suggesting a crucial role of short association fibers in AD-related decline of cognitive performance. Therefore, being one of the earliest events in the AD progression, demyelination might add to the factors that change covariance networks. Note that, by breaking down structural connectivity (in the absence of other factors, e.g., long-range connectivity, pathological processes coordinated across distributed brain regions, etc.) demyelination per se can lead only to *a decrease* in structural covariance. Considering the range of distance covered by U-fibers, a decrease in intra-lobar covariance can be expected.

This decrease is indeed what we have found here and illustrate with Fig. 3. However, the lost connections manifest both short-range (intralobar) and long-range (interlobar) covariance in all the ROIs except for the left temporal lobe. They likely indicate reduced mutual influences between distributed regions and/or *independent processes of local degeneration within frontal and parietal lobes* and/or population variants of AD with different topographies of degeneration. The newly gained connections are confined to the temporal and paralimbic regions. Similar changes were recently reported for VBM-based GM networks in mild-to-moderate^1^ AD patients (Yao et al. 2010). Such topography allows one to suggest that this hyper-covariance is due to a common AD-related pathological process, which strongly affects these areas already in early AD. Alternatively, the increase in the number of edges can be explained with remyelination. Indeed, the increased density of oligodendrocytes accompanies myelin loss in AD (Ihara et al. 2010). Remyelination processes may also underlie the AD-related increase in brain iron (Bartzokis 2011).

In our healthy elderly subjects, SWM hubs are predominantly distributed in the prefrontal, temporal, and posterior cingulate regions. In contrast to the GM networks (Buckner et al. 2009) and deep WM networks (Hagmann et al. 2008; van den Heuvel and Sporns 2011) with their hubs widely scattered across the association areas, we found no hubs in heteromodal parietal areas, but four hubs in the primary areas, which abound in short association fibers (Catani et al. 2012). Intriguingly, all the latter hubs together with many prefrontal ones were displaced in the mild AD patients. This fact might reflect that, while the primary areas are the last ones in the AD neurodegeneration sequence, their isolation from neighboring cortices is significant already in mild AD. The dramatically changed topography of the prefrontal hubs is consistent with the worsening of sustained attention, working memory, and executive functions in AD. Although the precise functions of the prefrontal U-fibers are yet to be clarified, their significant role in integrating the activity of local networks necessary for these functions is supported by their topography (Catani et al. 2012). In particular, the loss of hubs in BA 45 and 47 (reported here) against the background of significantly reduced myelin content (Fornari et al. 2012) might produce disturbance in semantic and memory functions due to the disconnection of these areas from the insula (Catani et al. 2012). The new hubs manifested by the AD group are mostly concentrated in the posterior areas of the left hemisphere and in the anterior and temporal areas of the right hemisphere. Since these changes in hub topography are not accompanied by an increase in edges in the corresponding regions (cf. Figs. 3 and 6), such a distribution can be explained by the relative preservation of SWM connections rather than by a compensatory response of the brain.

The topological properties of the MTR-based networks are only slightly changed, including a trend of a decreased clustering coefficient. This result is similar to the findings based on resting-state fMRI (Supekar et al. 2008) and MEG (Stam et al. 2009) networks in AD. Yet, in contrast to our finding, GM networks in AD show higher than normal clustering (He et al. 2008; Yao et al. 2010). The reason for this inconsistency may be different trajectories of degeneration of WM vs. GM in both mild AD and its likely precursor, amnesic MCI (Agosta et al. 2011; Carmeli et al. 2013). While such patients demonstrate widely distributed WM degeneration, their GM atrophy is mostly limited to the MTL.

Furthermore, GM networks show neither a significant interhemispheric asymmetry in healthy people (Mechelli et al. 2005), nor asymmetric involvement in the AD-related pathological process in patients (He et al. 2008; Yao et al. 2010).

### Interhemispheric asymmetry of SWM maps and networks in elderly controls and AD patients

A few MTI studies analyzed the interhemispheric asymmetry of the WM. Silver and colleagues did not find significant asymmetry of the regional WM, although MTR values in the left hemisphere were usually higher than in the homotopical regions of the right hemisphere (Silver et al. 1997). The results of Armstrong and colleagues (Armstrong et al. 2004) were less ambiguous: they reported a leftward MTR asymmetry for the whole hemisphere and regionally in the frontal and temporal lobes. Here we present the first attempt to show the interhemispheric differences in the SWM. In both groups we have found a rightward MTR asymmetry in the prefrontal regions and a leftward asymmetry in the posterior regions of AD patients, reminiscent of counter-clockwise cerebral torque (also known as “Yakovlevian”), which results from a combination of left-posterior and right-anterior hemispheric protrusions typical for a normal brain (Yakovlev and Rakic 1966; Good et al. 2001; Lancaster et al. 2003). According to Seldon's “balloon model”, the torque represents a broadening of the cortex in the right prefrontal cortex relative to the left, and in the left occipito–parieto–temporal cortex relative to the right, both due to a greater intracortical myelination, which moves cortical minicolumns apart, thereby stretching the cortex tangentially to the head surface (Seldon 2005). Our results suggest that indeed an asymmetry of myelination could be involved: at least U-fibers in these regions are asymmetrically myelinated as predicted by the hypothesis. Moreover, recently we found higher regional EEG synchronization in the left occipital and the right frontal locations relative to their interhemispheric counterparts – a finding also predicted by the “balloon model” (Jalili et al. 2010).

At a network level, we have found that normal elderly subjects demonstrate the strikingly asymmetric *intra*hemispheric patterns of regional covariance, with the strongly interconnected left hemisphere SWM accompanied by relatively low *inter*hemispheric covariance. Previous studies of structural GM covariance mostly showed symmetric networks (e.g., Mechelli et al. 2005; Wu et al. 2012). Furthermore, the pattern of SWM network lateralization is dissimilar from the pattern of interhemispheric MTR differences in our subjects. In contrast, Kang et al. (2011) found leftward perisylvian asymmetries in fractional anisotropy (FA) and MTR of the SWM in young healthy subjects. Phillips et al. (2013), working with a population of 18- to 74–year-old subjects, observed widely distributed leftward FA asymmetry of SWM in all the brain lobes and averaged across each hemisphere. Considering that subjects in our sample were older than those in cited papers, a discrepancy between these findings suggests a reduction in interhemispheric SWM asymmetry late in life. It seems to be supported by behavioral, fMRI activation, and fMRI connectivity studies, which repeatedly showed an asymmetry decline in elderly people (Cabeza 2002; Li et al. 2009), although significant age-by-asymmetry effects were not observed for the SWM in a recent study (Phillips et al. 2013).

What are the factors that cause the network asymmetry found here? These might be special features of U-fibers in the left hemisphere, e.g., their relatively long length or dense branching compared to the opposite hemisphere. Although we failed to find any postmortem observations on asymmetry of human U-fibers in the literature, there do exist data about the asymmetry of intracortical connectivity (Galuske et al. 2000). These authors studied the interhemispheric differences in the intrinsic microcircuitry of BA 22 (Vernicke's area in the left hemisphere) and found that tangential connections ranging several millimeters are about 20% longer in the left hemisphere.

Another likely factor for increased structural covariance in the left hemisphere could be asymmetries in the deep WM fibers, which implement experience-dependent interregional influences through functional coupling of distributed areas, propagate metabolites, etc. Indeed, many studies showed leftward lateralization of the arcuate fasciculus that connects the posterior temporal and the inferior frontal cortices (Concha et al. 2012, Nucifora et al. 2005; Takao et al. 2011a). A significantly higher FA for most parts of the left cingulum, a prominent fiber tract connecting parts of the paralimbic system, is also well documented (Gong et al. 2005, Takao et al. 2011a). Moreover, an analysis with tract-based spatial statistics of the WM in 857 normal subjects aged between 24 and 85 years showed stable leftward asymmetry of FA (Takao et al. 2011b).

## Conclusion

A comparison of the MTR-based myelination maps and MTR-based networks in healthy aged people and mild AD patients suggests that some network properties can be explained through the myelination topography and its changes in AD. Specifically, the distribution of lost edges and changed hub locations generally recapitulate the topography of demyelination in AD. At the same time, there are network properties that cannot be clarified by the myelination maps or their changes and allow us to suggest some new features of the pathological process. First, in early AD, coordinated degeneration is characteristic for the MTL and paralimbic regions, whereas other association areas show discoordinated degenerative processes. Alternatively, this finding might point to the variants of AD that are implicitly present in our AD group, e.g., early-onset and late-onset AD. Second, the interhemispheric asymmetry of structural covariance seems to have no reflection in the interhemispheric asymmetry of myelination in either group. This asymmetry also differentiates SWM networks from mostly symmetrical GM networks, thus suggesting specific developmental and/or plasticity factors that affect only the SWM. Therefore, the first attempt of a graph analysis of SWM networks has resulted in new and partially intriguing findings, which are of interest for clinical application while requiring replication in a larger subject group.

## Footnotes

1 In their AD group MMSE was 22.41 ± 3.40 vs. 28.89 ± 1.13 in the control group (Z. Yao, pers. comm.).

## Supporting Information

**Data S1**. Computation of the hippocampal volume.
