# Supplementary material for: Structural covariance of superficial white matter in mild Alzheimer's disease compared to normal aging
Source: Brain Behav. 2014 Jul 28;4(5):721–37. doi: 10.1002/brb3.252 (PMC4113976; doi:10.1002/brb3.252)
Supplement: Supplementary file 1 — Data S1. Computation of the hippocampal volume. [file brb30004-0721-SD1.docx]

***Computation of the hippocampal volume.*** In the MNI space, using the AAL atlas (Tzourio-Mazoyer et al., 2002), we obtained a mask of the two hippocampi, which was then projected into a subject’s space through inverse deformation fields (SPM8 at http://www.fil.ion.ucl.ac.uk/spm/). The individual volume of the hippocampus corresponds to a total of the voxels extracted via the subject-specific mask. Between-group differences were evaluated within the framework of the GLM with а permutation test using 10000 permutations. Age, gender, and total intracranial volume served as covariates.

To further corroborate this result, we ran a standard voxel-based morphometry analysis in SPM8. Segmentation was performed with the unified segmentation algorithm (Ashburner and Friston, 2005), co-registration with the DARTEL algorithm (Ashburner, 2007), and statistical inference with Random Field Theory (Friston et al., 2005) on the modulated and smoothed (6mm FWHM) GM images.

**References**

Ashburner J, Friston KJ (2005) Unified segmentation. Neuroimage 26: 839–851.

Ashburner J (2007) A fast diffeomorphic image registration algorithm. Neuroimage 38:95-113.

Friston KJ, Holmes AP, Worsley KJ, Poline JB, Frith CD, et al. (1995) Statistical parametric maps in functional imaging: a general linear approach. Hum Brain Mapp 2: 189-210.

Tzourio-Mazoyer N, Landeau B, Papathanassiou D, Crivello F, Etard O, et al. (2002) Automated anatomical labeling of activations in SPM using a macroscopic anatomical parcellation of the MNI MRI single-subject brain. Neuroimage 15: 273-289.
